# Site-specific fracture risk in chronic hypoparathyroidism: systematic review and meta-analysis

**DOI:** 10.1007/s11154-026-10051-8

**Published:** 2026-05-11

**Authors:** Andrea Palermo, Eleonora Sargentini, Roberto Cesareo, Gaia Tabacco, Anda Mihaela Naciu, Pierpaolo Trimboli

**Affiliations:** 1 Unit of Endocrinology and Diabetes, Campus Bio-Medico University, Rome, Italy; 2 Unit of Metabolic Bone and Thyroid Disorders, Fondazione Policlinico Campus Bio-Medico, Rome, Italy; 3 UOS Malattie Metaboliche, Santa Maria Goretti Hospital, Latina, Italy; 4 Department for the Promotion of Human Science and Quality of Life, San Raffaele Open University, Rome, Italy; 5 Thyroid Unit, Clinic for Endocrinology and Diabetology, Ente Ospedaliero Cantonale (EOC), Bellinzona, Switzerland; 6 Faculty of Biomedical Sciences, Università Della Svizzera Italiana, Lugano, Switzerland

**Keywords:** Fracture, Hypoparathyroidism, PTH, Vertebral fracture, Hip fracture

## Abstract

Chronic hypoparathyroidism (HypoPT) is characterized by sustained parathyroid hormone (PTH) deficiency, leading to reduced bone turnover, increased bone mineral density (BMD), and altered skeletal microarchitecture. Despite preserved or elevated BMD, fracture risk in HypoPT remains controversial. We conducted a systematic review and meta-analysis to assess site-specific fracture risk and the influence of disease duration in patients with chronic HypoPT. Fifteen observational studies, including 13,563 HypoPT subjects and 137,245 controls, were analyzed. HypoPT subjects had a significantly higher risk of vertebral fractures (VFs) than controls (OR 1.88, 95% CI 1.23–2.88), with the excess risk confined to individuals with non-surgical disease (OR 3.05, 95% CI 1.68–5.54). No significant difference between HypoPT and controls were observed in non-vertebral (OR 1.01 (95% CI 0.83, 1.22) or hip fractures (OR 0.81, 95% CI 0.63–1.04). Meta-regression analyses demonstrated that longer median disease duration was associated with higher fracture prevalence at non-vertebral and hip sites. These findings support a site-specific pattern of skeletal fragility, characterized by increased vertebral vulnerability, particularly in non-surgical HypoPT, alongside relative protection at cortical-predominant sites. Fracture risk in HypoPT appears to evolve over time and to become evident during long-term follow-up. Longitudinal, site-directed fracture assessment should be considered in clinical practice.

**Supplementary Information:**

The online version contains supplementary material available at https://doi.org/10.1007/s11154-026-10051-8.

## Introduction

Hypoparathyroidism (HypoPT) is a rare endocrine disorder characterized by hypocalcemia due to absent or inappropriately low secretion of parathyroid hormone (PTH) [1]. The condition most commonly arises as a complication of anterior neck surgery, accounting for approximately 70–80% of cases, while non-surgical forms include autoimmune, genetic, and idiopathic etiologies. Chronic HypoPT requires lifelong management with calcium supplementation and active vitamin D analogues that represent its first line therapy; however, PTH replacement therapy is emerging as a promising option and may play a broader role in selected patients in the future [1].

PTH plays a pivotal role in skeletal homeostasis by regulating calcium–phosphate metabolism and bone remodeling. Through stimulation of osteoblast-lineage cells, PTH promotes Receptor Activator of Nuclear factor Kappa-B Ligand (RANKL) expression and osteoclast activation, thereby maintaining physiological bone turnover [1]. In chronic HypoPT, sustained PTH deficiency leads to a profound suppression of bone remodeling, resulting in a state of low bone turnover. Histomorphometric and imaging studies consistently demonstrate increased trabecular and cortical bone mass, elevated volumetric bone mineral density, and reduced bone turnover markers [1]. However, despite this apparent increase in bone quantity, bone quality is altered. Increased mineralization, reduced remodeling dynamics, and abnormalities in microarchitecture may render bone more brittle, potentially compromising skeletal strength, particularly at trabecular-rich sites such as the spine [1].

Consistent with these pathophysiological mechanisms, clinical data on fracture risk in HypoPT have been conflicting. Several observational studies have reported preserved or even reduced risk of non-vertebral fractures (VFs) [2], whereas others have identified an increased prevalence of VFs [3–5] and upper extremities fractures [6] despite normal or elevated bone mineral density (BMD). These discrepancies are likely driven by heterogeneity in disease etiology (surgical versus non-surgical), duration of HypoPT, fracture ascertainment methods, and population characteristics.

To date, only one systematic review and meta-analysis has specifically addressed fracture risk in HypoPT [7]. This meta-analysis demonstrated a nearly twofold increased risk of VFs, confined to patients with non-surgical HypoPT, while no increased risk was observed for hip, humeral, or overall fractures. In contrast, postsurgical HypoPT was not associated with a statistically significant increase in VFs risk, leading to the hypothesis that disease chronicity, rather than etiology per se, may be a critical determinant of skeletal fragility.

More recently, large population-based retrospective cohort studies have provided new insights into fracture patterns in chronic HypoPT [8–10]. Nationwide registry data have shown that patients with chronic HypoPT exhibit a significantly increased risk of VFs, alongside a reduced risk of femoral fractures, when compared with matched controls. Importantly, these studies suggest that VFs risk becomes more apparent with longer disease duration, supporting the concept that prolonged PTH deficiency progressively impairs trabecular bone quality, even in the absence of overt bone loss [10]. Notably, this increased VFs risk has been observed not only in non-surgical HypoPT but also in post-surgical cases with long-standing disease, challenging earlier assumptions derived from smaller cohorts.

Taken together, the available evidence suggests a site-specific and time-dependent effect of chronic HypoPT on fracture risk, characterized by relative protection at cortical sites and increased vulnerability at the spine. However, existing studies differ substantially in design, population size, follow-up duration, and fracture assessment, and the modifying role of disease duration has not been systematically evaluated.

Therefore, the present meta-analysis aims to comprehensively assess fracture risk in patients with chronic HypoPT, with a specific focus on fracture site and influence of disease duration. By integrating data from population-based studies and exploring heterogeneity through meta-regression analyses, this work seeks to clarify whether prolonged exposure to PTH deficiency selectively increases VFs risk and to reconcile previously conflicting findings in the literature. In particular, the primary endpoint of the study was to quantify the risk of vertebral, hip, and non-vertebral fractures in patients with hypoparathyroidism compared with controls. Additionally, the overall risk of fracture at any site was estimated by pooling data from studies reporting fractures at all anatomical locations.

## Materials and methods

### Review conduct

This meta-analysis was conducted and reported according to the Preferred Reporting Items for Systematic Reviews and Meta-analyses (PRISMA) 2020 statement [11]. The study protocol was registered with PROSPERO (CRD420261295028). This study is a systematic review and *meta*-analysis of previously published data and did not involve any new studies with human participants or animals performed by any of the authors. Therefore, Institutional Review Board approval was not required.

### Search strategy

Two investigators (ES and AMN) independently conducted a systematic literature search in the PubMed, Scopus, and Web of Science databases from inception to January 25, 2026 (last search date). The search strategy combined controlled vocabulary terms, where applicable, and free-text keywords related to HypoPT and fractures, using appropriate Boolean operators. The main search terms included “hypoparathyroidism” AND “fractures.” The search was restricted to studies published in the English language. In addition, the reference lists of relevant reviews and all eligible articles were manually screened to identify further potentially relevant studies. When required data were missing or unclear, corresponding authors of the original studies were contacted to obtain additional information.

### Study eligibility and exclusion criteria

Eligibility criteria were predefined according to study population, comparator, outcomes, and study design. Eligible studies were observational, including prospective or retrospective cohort and case–control studies. Studies were required to include adult patients with chronic HypoPT, either postsurgical or non-surgical, and a comparison group of individuals without HypoPT. To be eligible, studies had to report fracture outcomes, defined as the occurrence of clinical and/or morphometric fractures, and to provide sufficient data to derive fracture event counts or effect estimates for HypoPT patients and controls. Studies were excluded if they included patients with pseudohypoparathyroidism, functional HypoPT, normocalcemic HypoPT, or autoimmune polyendocrinopathy–candidiasis–ectodermal dystrophy (APECED), unless data for chronic HypoPT could be clearly distinguished. Additional study exclusion criteria were the absence of a non-hypoparathyroid control group, lack of reported fracture outcome data, or publication as reviews, editorials, comments, letters to the editor, or case reports.

### Data extraction

Two investigators (ES and AMN) independently screened titles and abstracts to remove duplicate records and to exclude studies that did not meet the predefined eligibility criteria. Full texts of potentially eligible studies were subsequently retrieved and assessed for inclusion. Any disagreements at any stage of the selection process were resolved through discussion and consensus or, when necessary, by consultation with a third senior investigator (GT).

Data extraction was performed independently by the same two investigators using a predefined data extraction form. Extracted data included study characteristics, HypoPT etiology (nonsurgical and/or postsurgical), number of patients with HypoPT, number of non-hypoparathyroid control subjects, fracture site (any fracture, VFs, non-vertebral or hip), fracture type (clinical or morphometric), and the number of fracture events reported in HypoPT patients and controls.

For the purpose of quantitative synthesis, when available, fracture outcomes were preferentially extracted as the number of patients experiencing at least one fracture rather than the total number of fracture events. This patient-level approach was adopted to minimize potential within-subject clustering effects and to avoid overestimation of risk due to individuals sustaining multiple fractures.

When this distinction was unclear, analyses assumed that the number of fractures equaled the number of affected patients.

### Assessment of study quality and risk of bias

The methodological quality and risk of bias of the included studies were independently assessed by two co-authors (AMN and GT) using the National Heart, Lung, and Blood Institute Quality Assessment Tool for Observational Cohort and Cross-Sectional Studies.

The tool evaluates key methodological domains, including clarity of the research question, eligibility and selection criteria, sample size justification, exposure definition and assessment, outcome definition and measurement, control of confounding variables, completeness of follow-up, and blinding of outcome assessors. Each domain was rated as low risk of bias, high risk of bias, or not reported, in accordance with guidance from the Cochrane Handbook for Systematic Reviews of Interventions.

### Statistical analysis

Proportion meta-analyses were performed to estimate the pooled prevalence of fractures among patients with HypoPT and their etiological subgroups, and control subjects. Odds ratio (OR) meta-analyses were conducted to quantify the relative risk of fracture in HypoPT patients compared with controls. All analyses were patient-based to avoid biases deriving from fracture-bases estimates that were likely to overestimate the effect.

Random-effects models were systematically applied to account for expected clinical and methodological heterogeneity across studies. Pooled estimates were presented with 95% confidence intervals (95% CI) and displayed in forest plots. Statistical heterogeneity was assessed using the I^2^ statistic, with values > 50% considered indicative of substantial heterogeneity.

When heterogeneity was identified, potential sources were explored using meta-regression analyses for continuous variables and subgroup analyses for categorical variables. For meta-regression, disease duration was operationalized as the median follow-up time reported in each study. In postsurgical HypoPT, this corresponded to the time elapsed since the surgical procedure leading to HypoPT or, when explicitly reported, the duration of study observation. In non-surgical HypoPT, disease duration was defined as the time since diagnosis or, if unavailable, the study follow-up period. When both measures were reported, the median duration was preferentially extracted.

Given the inherent differences in disease onset between surgical and non-surgical forms, duration was interpreted as a proxy of cumulative exposure to PTH deficiency rather than as an exact measure of biological disease chronicity. A *p*-value < 0.05 was considered statistically significant in meta-regression analyses. Differences between subgroups were inferred when 95% confidence intervals did not overlap. All statistical analyses were performed using OpenMeta[Analyst] (Center for Evidence Synthesis in Health, Brown University).

## Results

### Study selection

**Fig. 1:**
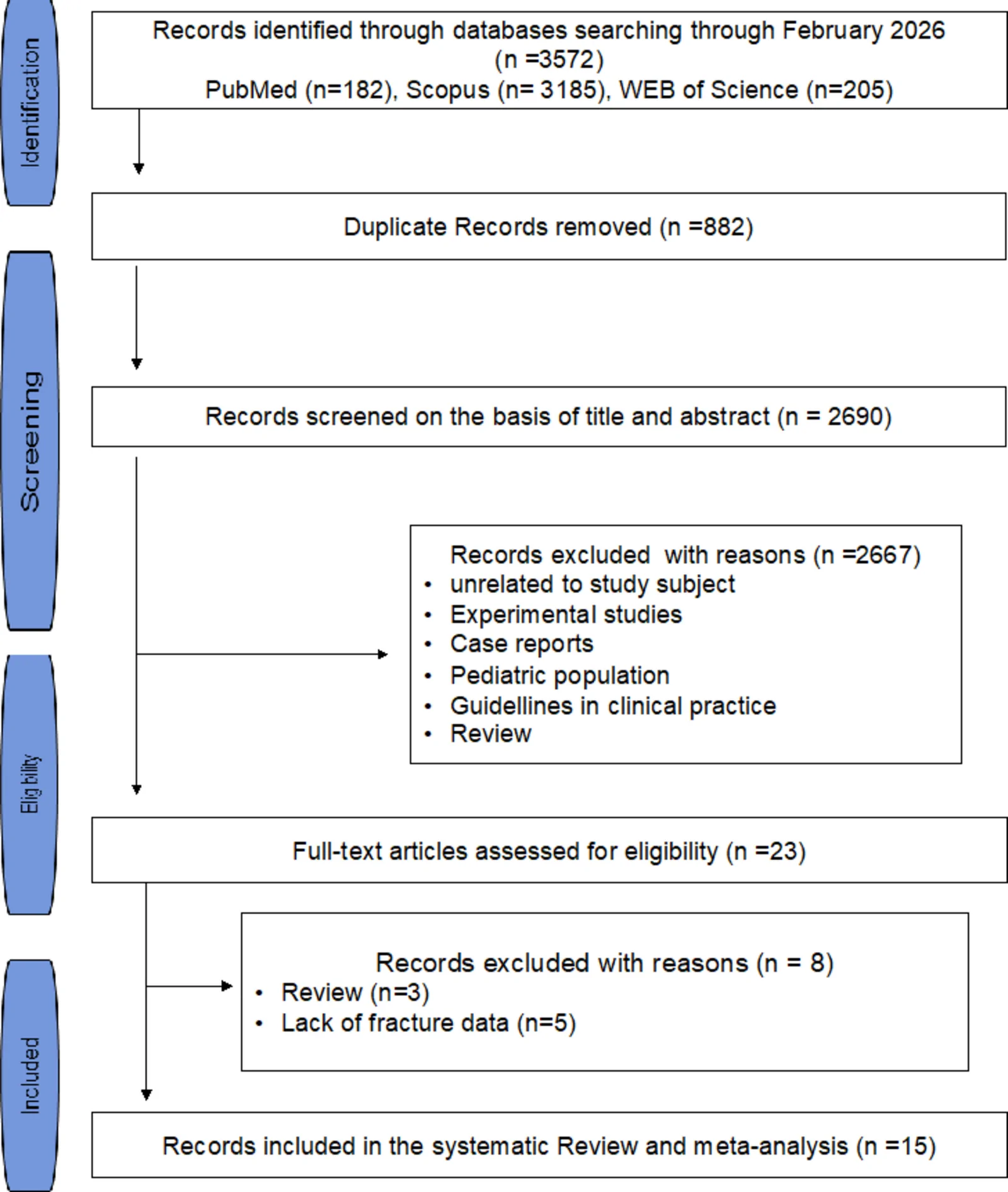
PRISMA flowchart. Diagram of search flow

A total of 3572 records were initially identified through the search strategy. After removing duplicates and screening the titles and abstracts, 23 articles were retrieved for full-text review. Of these, 23 studies were excluded due to either unclear or inconsistent data regarding fracture data (*n* = 5) or because they were reviews (*n* = 3). Ultimately, 15 studies were included in this systematic review. The study selection process has been summarized in the PRISMA flowchart (Fig. 1).

### Study quality assessment

The risk of bias is detailed in the Supplemental Material Table S1. Overall, the methodological quality of the included studies was predominantly moderate. 10 studies were rated as fair quality and 5 as poor quality. The most common sources of potential bias were lack of sample size justification, inadequate control for confounding variables, and absence of blinding of outcome assessors.

### Qualitative analysis (systematic review)

The 15 studies included in this systematic review were published between 2013 and 2025. All were observational in design and based on retrospective analyses performed after case enrollment. Most were multicenter or population-based studies, whereas six were conducted at a single center. Regarding geographical distribution, six studies were conducted in Europe (Italy [*n* = 1], Sweden [*n* = 1], Denmark [*n* = 2], and the UK [*n* = 2]), six in Asia (China [*n* = 1], South Korea [*n* = 3], and India [*n* = 2]), two in Latin America (both in Brazil), and one in the Middle East (Israel).

Overall, the 15 observational studies included in the meta-analysis comprised 13,563 patients with HypoPT and 137,245 controls without HypoPT (Table 1). Eight studies focused on postsurgical HypoPT populations, whereas four evaluated exclusively nonsurgical HypoPT. In particular, regarding non-surgical HypoPT, most of the included studies predominantly enrolled subjects with idiopathic HypoPT, while less frequent etiologies included autosomal dominant hypoparathyroidism (ADH), DiGeorge syndrome, and autoimmune polyglandular failure. Vadiveloo included both postsurgical and nonsurgical cases, but in the statistical analysis we considered the two groups individually [12]. Slutzky-Shraga also enrolled both subgroups; however, for the purposes of our meta-analysis, the nonsurgical subgroup was excluded due to the young age of the population and the lack of information on fractures [13]. In one study the type of HypoPT was not specified, and no distinction was made between the non-surgical and post-surgical forms [10].

**Table 1:** Human studies on Fractures in Hypoparathyroidism included in the systematic review and meta-analysis

First author, year [ref]	Study type and place of study	Study population (number of patients/age)	Fracture sites	Number of subjects with fractures				Comments
				Total	VFs	NVFS	HFx	
Post-surgical HypoPT								
Ahn, 2023 [8]	Retrospective cohort, Korea	8789 HypoPT, age NA; 107032ctrls, age NA	VFs (clinical), NVFS (humerus, wrist), HFx	159 HypoPT; 2390 ctrls	36 HypoPT; 613 ctrls	121 HypoPT; 1746 ctrls	6 HypoPT; 95 ctrls	Possible underestimation of asymptomatic VFs; administrative definition of HypoPT
Ku, 2025 [9]	Retrospective cohort, Korea	417 HypoPT, age 54y; 827 ctrls, age 54y	VFs (clinical), NVFS (humerus, wrist), HFx	31 HypoPT; 28 ctrls	15 HypoPT; 8 ctrls	14 HypoPT; 17 ctrls	2 HypoPT; 3 ctrls	Possible misclassification due to definitions based on administrative (claims) data
Mendonça, 2013 [3]	Observational cross-sectional case–control study, Brazil	16 HypoPT, age 62.3y; 17 ctrls, age 58y	VFs (morphometric)	10 HypoPT; 2 ctrls	10 HypoPT; 2 ctrls	NA	NA	Small number of patients; fractures other than VFs were not assessed
Vadiveloo, 2018[12]	Observational retrospective population-based study, UK	116 HypoPT, age 47.1y; 1301 ctrls, age 49.6y	NA (assumed clinical)	10 HypoPT; 71 ctrls	NA	NA	NA	Small number of patients; inability to distinguish fracture types; no data on BMD
Cipriani, 2021 [4]	Observational cross-sectional case control study, Italy	50 HypoPT, age 65.4y, 40 ctrls 64.2y	VFs (morphometric)	10 HypoPT; 3 ctrls	8 HypoPT; 3 ctrls	2 HypoPT; 0 ctrls	0 HypoPT; 0 ctrls	Low number of fracture events, limited ability to detect additional risk factors, uncertainty about whether VFs occurred before HypoPT diagnosis, and limited generalizability since only post-surgical chronic HypoPT patients were studied
Underbjerg, 2014 [2]	Observational case-finding study	688 HypoPT, age 49y, 2064 ctrls49y	VFs (clinical), NVFS (pelvic fractures, proximal femur fractures, atypical femur fractures, forearm fractures, proximal humerus, ancle fracture incl. the foot, other upper extremities fracture, other lower extremities fracture, other types of fracture), HFx	102 HypoPT; 305 ctrls	13 HypoPT; 36ctrls	76 HypoPT; 218 ctrls	13 HypoPT; 51ctrls	Sample size was small and key variables were missing (e.g., bisphosphonate use in controls; non–post-surgical HypoPT), limiting detection of rare events Registry undercaptures mild/GP-managed conditions and possible differential specialist access may bias comparisons
Slutzky-Shraga, 2023 [13]	Retrospective, single-center observational study	105 HypoPT, age 47y, 142 ctrls47y	VFs (clinical and morphometric both), NVFS(humerus, pelvis, ankle, wrist, ribs), HFx	32 HypoPT; 30 ctrls	16 HypoPT; 21ctrls	12 HypoPT; 6ctrls	4 HypoPT; 3 ctrls	Retrospective single-center study with possible selection bias and few fracture events, limiting power Treatment details and key clinical data (HypoPT duration/etiology, bone turnover markers) were missing, and asymptomatic VFs may be underestimated
Lui, 2021 [14]	Retrospective observational cohort study	460 HypoPT, age 49y, 3663 ctrls49y	VFs (clinical), NVFS (upper extremities, lower extremities)	13 HypoPT; 113 ctrls	1 HypoPT; 15 ctrls	12 HypoPT; 91ctrls	NA	Lack of data on BMD and bone turnover markers
d’Alva, 2022 [18]	Case–control observationalstudy	40 HypoPT, age 58.7y, 40 ctrls58.7y	VFs (clinical and morphometric both), NVFS(wrist, foot, patella), HFx	5 HypoPT; 3 ctrls	5 HypoPT; 3 ctrls	2 HypoPT; 3 ctrls	0 HypoPT; 0 ctrls	Small sample size (limited statistical power), half of the controls were hospital staff (potential healthy-worker bias), lack of BMD data and VFs assessment, and missing 25(OH)D measurements in controls
Reddy, 2025 [16]	Retrospective observational matched cohort study	215 HypoPT, age 53.9y, 2149 ctrls53.9y	VFs (clinical), NVFS (wrist, tibia, fibula, femur, radius, ulna, humerus, pelvis and ribs), HFx	8 HypoPT; 70ctrls	8 HypoPT; 70 ctrls	NA	NA	Retrospective registry/EHR design with possible misclassification and limited clinical detail (severity/biochemical control, treatment specifics); relatively small HypoPT cohort with few events for some outcomes and reduced power; potential residual confounding and surveillance/detection bias
Non-surgical HypoPT								
Chawla, 2017 [17]	Observational cohort, India	104 HypoPT, age 37.2y; 64 ctrls, age 37.53y	VFs (morphometric)	19 HypoPT; 3 ctrls	19 HypoPT; 3 ctrls	NA	NA	Microarchitectural changes in bone were not assessed
Kim, 2020 [15]	Observational retrospective cohort study, Korea	210 HypoPT, age 39.2y; 2075 ctrls, age 39.2y	VFs (clinical), NVFS (humerus, wrist), HFx	15 HypoPT; 116 ctrls	9 HypoPT; 39 ctrls	7 HypoPT; 71 ctrls	0 HypoPT; 11 ctrls	Diagnostic data (not collected for research purposes) may not accurately estimate disease prevalence; therefore, prescription data and exclusion variables were added to improve accuracy
Vadiveloo, 2018 [12]	Observational retrospective population-based study, UK	106 HypoPT, age 50.5y; 1301 ctrls, age 49.6y	NA	9 HypoPT; 71 ctrls	NA	NA	NA	Small number of patients; inability to distinguish fracture types; no data on BMD
Underbjerg, 2015 [6]	Observationalcase-findingstudy	180 HypoPT, age 49.7y; 540 ctrls, age 49.7y	VFs (clinical), NVFS (pelvic fractures, proximal femur fractures, atypical femur fractures, forearm fractures, proximal humerus, ancle fracture incl. the foot, other upper extremities fracture, other lower extremities fracture, other types of fracture), HFx	34 HypoPT; 70ctrls	5 HypoPT; 11 ctrls	20 HypoPT; 44 ctrls	9 HypoPT; 15 ctrls	Few events in some complication subgroups limit statistical power to estimate specific risks NS-HypoPT prevalence is likely underestimated: reported figures are minimum estimates, and undiagnosed cases may exist (e.g., autosomal dominant hypocalcaemia, DiGeorge syndrome)
Saha, 2021 [5]	Observational cross-sectional, matched case–control study	152 HypoPT, age 40.2y; 152 ctrls, age 39y	VFs (morphometric)	47 HypoPT; 12 ctrls	47 HypoPT; 12 ctrls	NA	NA	Cross-sectional, single-center design limits causality and generalizability; clinical fractures were few and mostly morphometric. Bone turnover dynamics, histomorphometry, and the effects of long-term therapies/comorbidities were not fully assessed, potentially underestimating risk in poorer-controlled patients
Both Post-surgical and Non-surgical HypoPT*								
Björnsdottir, 2025 [10]	Observationalcohort study	1915 HypoPT, age 53.9y; 15,838 ctrls, age 50.2 y	VFs (clinical), NVFS (pelvic, proximal humerus, forearm), HFx	181 HypoPT; 1707 ctrls	49 HypoPT; 267 ctrls	93 HypoPT; 980 ctrls	39 HypoPT; 460ctrls	Limited clinical details (e.g., BMD and treatment specifics), with possible underestimation of non-clinical fractures

*Abbreviations:* *BMD* bone mineral density; *ctrls* controls; *F* female; *HFx* hip fractures; *HypoPT* hypoparathyroidism; *M* male; *m* months; *NA* not available; *NS* non-significative; *NVFS* non-vertebral fractures; *TFx* total fractures; *VFs* vertebral fracture; *y* years

*in those studies, it was impossible to distinguish the number of fractures accordingly to HypoPT subtypes

Clinical fractures were reported in nine studies [2, 6, 8–10, 12, 14–16] whereas only morphometric vertebral fractures were assessed in four studies [3–5, 17]. Slutzy-Shraga and D’Alva assessed both clinical and morphometric fractures [13, 18]. Two studies [6, 14] included also patients treated with PTH analogues. Specifically, Underbjerg reported four patients receiving PTH (1–84) [6]. In Lui’s study, the use of anti-osteoporosis medications, including teriparatide, was collected among baseline covariates and was not an exclusion criterion; however, the exact number of HypoPT patients receiving teriparatide was not specified [14]. In contrast, Cipriani, Mendonça, and Björnsdottir [3, 4, 10] explicitly excluded patients treated with PTH analogues. Data on BMD were reported in only six studies [3–5, 8, 13, 17]. In all studies, control subjects were matched for age and sex. In Ahn and Ku studies it was mentioned only the number of fracture events, instead of the number of fractured subjects; thus, we assumed that the number of events corresponded to the number of fractured subjects [8, 9]. The results of the meta-analysis are presented according to the anatomical site at which fractures were reported (Table 1).

### Quantitative analysis

#### Risk of any fracture

Twelve studies [2, 4, 6, 8, 9, 12–16, 18] including 13,291 patients with HypoPT and 137,012 controls, reported data on fractures at any site. The total number of HypoPT with any fracture was 609/13,291, while the number of controls with any fracture was 4977/137,012. The pooled prevalence of fractures among HypoPT patients was 10.0% (95% CI 6.9–13.1; I^2^ = 96.38%), while in the control group it was 7.4% (95% CI 5.1–9.8; I^2^ = 99.24%).

Overall, pooled analysis did not demonstrate a statistically significant difference in the risk of fractures at any site between HypoPT patients and controls (OR 1.20, 95% CI 0.99–1.47; I^2^ = 64.18%). Meta-regression analysis using disease duration as a covariate did not reveal a significant association (*p* = 0.203).

**Fig. 2:**
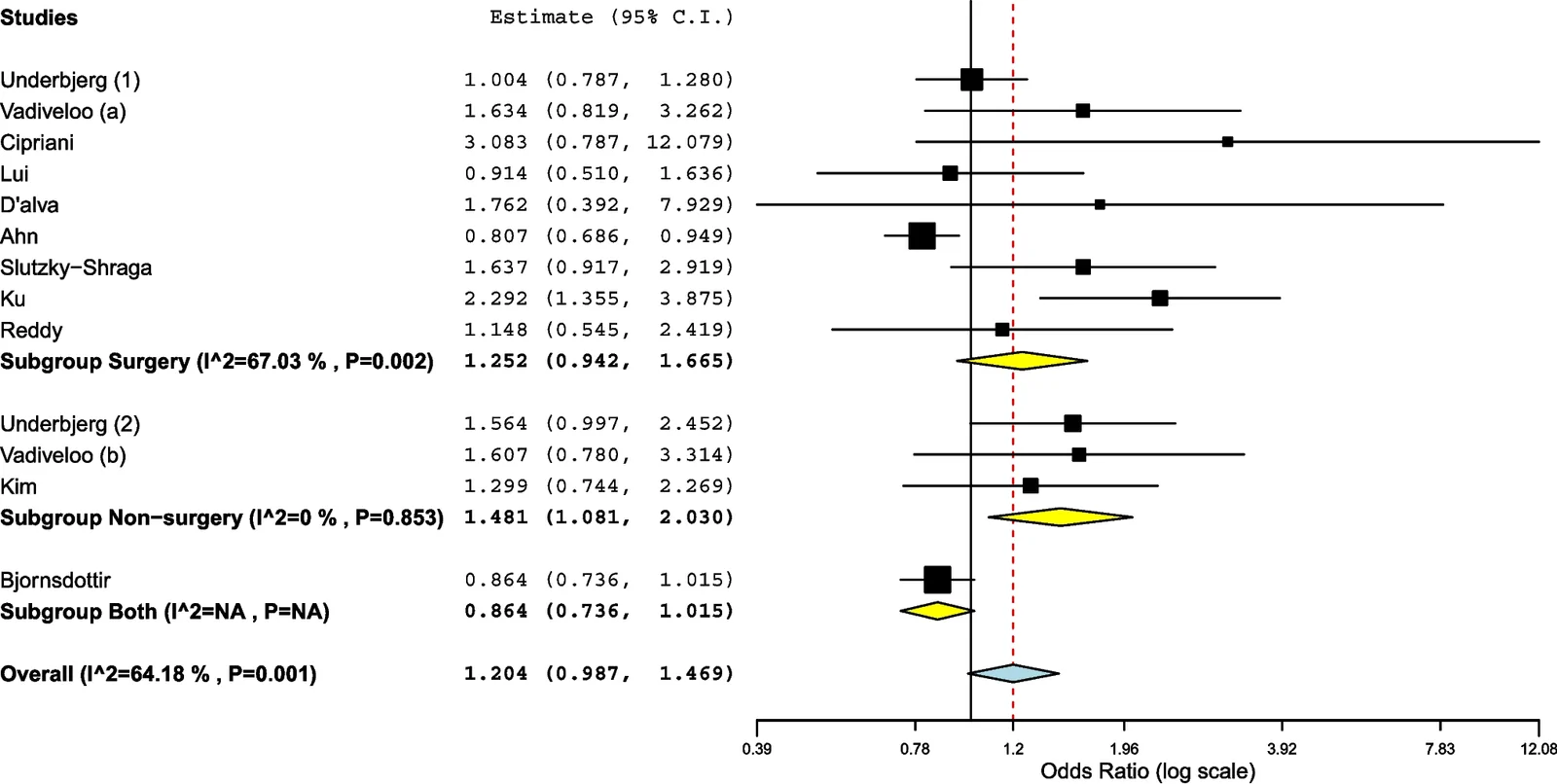
Any fracture: OR comparing HypoPT and controls. Blue diamond indicates the pooled OR in all included studies and its wideness indicates 95%CI. Yellow diamonds indicate the pooled OR in subgroups. Black square and black line of any series indicate its weight and 95% CI, respectively

However, subgroup analysis showed that patients with non-surgical HypoPT was significantly associated with increased risk of any fracture compared to controls (OR 1.48, 95% CI 1.08–2.03), without heterogeneity (I^2^ = 0%; p for heterogeneity = 0.853) (Fig. 2).

#### Risk of vertebral fracture

Thirteen studies [2–6, 8–10, 13–15, 17, 18] investigated VFs, including a total of 13,126 patients with HypoPT and 132,494 controls. The total number of HypoPT with VFs was 233/13,126, while the number of controls with VFs was 1033/132,494. The pooled prevalence of vertebral fractures was 4.6% (95% CI 3.1–6.1; I^2^ = 93.84%) in HypoPT patients and 1.6% (95% CI 1.0–2.1; I^2^ = 93.98%) in controls. Among HypoPT patients, the pooled fracture rate was significantly lower in the post-surgical subgroup (2.3%, 95% CI 1.0–3.5) compared with the non-surgical subgroup (13.3%, 95% CI 4.3–22.3).

**Fig. 3:**
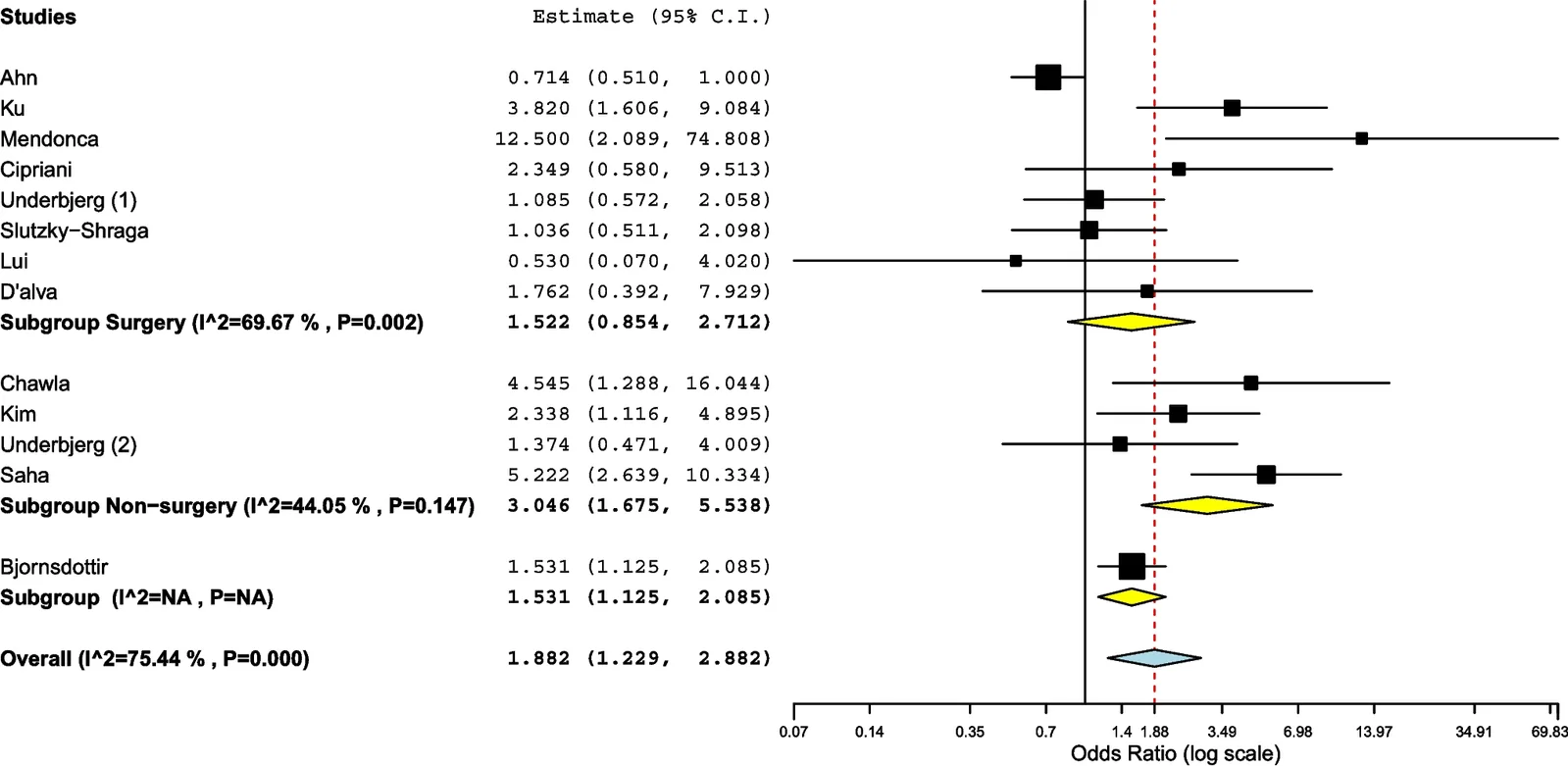
Vertebral fractures: OR comparing HypoPT and controls. Blue diamond indicates the pooled OR in all included studies and its wideness indicates 95%CI. Yellow diamonds indicate the pooled OR in subgroups. Black square and black line of any series indicate its weight and 95% CI, respectively

Overall, pooled analysis showed that HypoPT was associated with increased risk of VFs compared to controls (OR 1.88, 95% CI 1.12–2.88; I^2^ = 75.44%). Subgroup analysis demonstrated that this increased risk was confined to patients with non-surgical HypoPT (OR 3.05, 95% CI 1.68–5.54; I^2^ = 44%), whereas no significant association was observed in those with post-surgical HypoPT (OR 1.52, 95% CI 0.85–2.71) (Fig. 3).

#### Risk of non-vertebral fracture

**Fig. 4:**
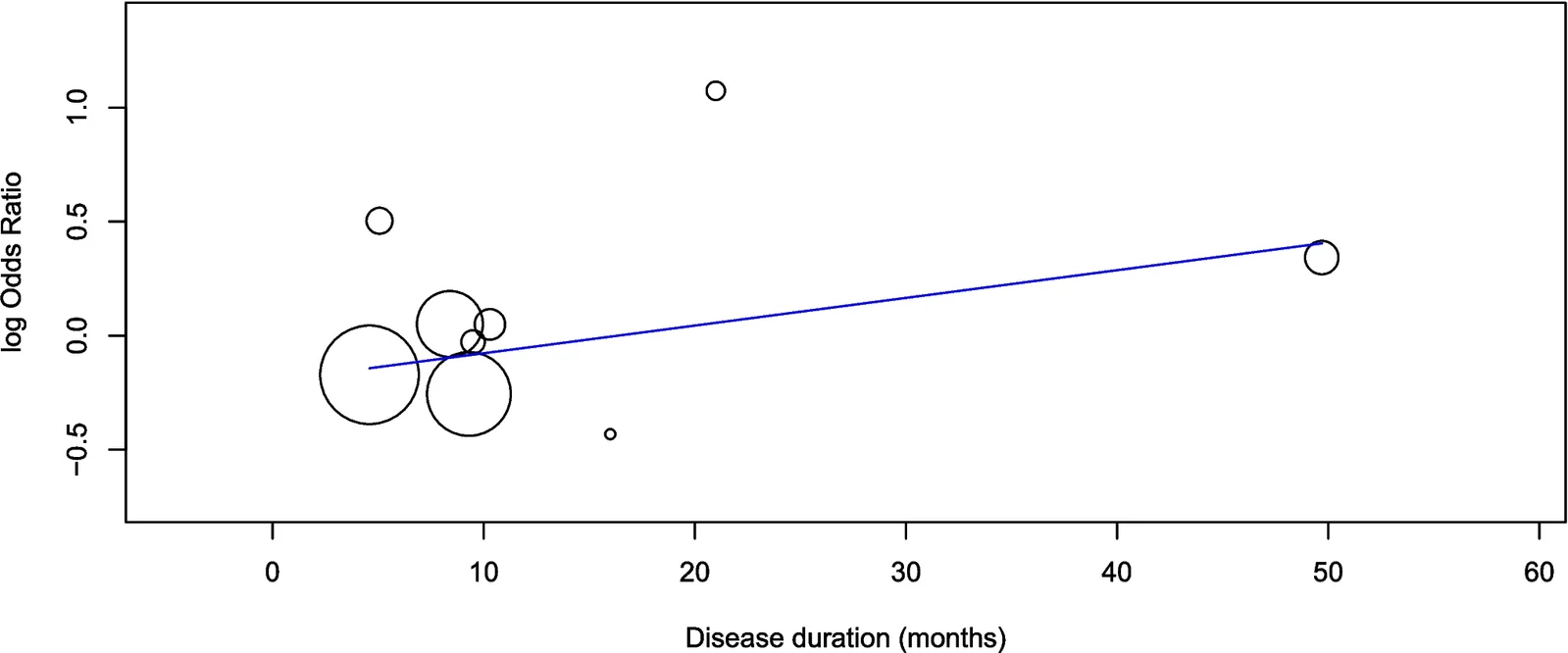
Non-vertebral fractures: meta-regression analysis exploring OR comparing HypoPT and controls according to disease duration. Any circle identifies one series, and its size differs according to the series weight

Ten studies [2, 4, 6, 8–10, 13–15, 18] analyzed the percentage of non-vertebral fracture in a total of 12,854 HypoPT patients and 132,261 controls. The total number of HypoPT with non-vertebral fractures was 359/12,854, while the number of controls with non-vertebral fractures was 3176/132,261. The pooled proportion of non-vertebral fracture in the former group was 0.055 (95% CI 0.031–0.078) with I^2^ 92.13%. This heterogeneity was explored by meta-regression analysis showing that longer median disease duration may be associated with a higher fracture prevalence (*p* = 0.06) (Fig. 4).

The proportion of non-vertebral fracture in control group was 0.046 (95% CI 0.028–0.064) with I^2^ 98.82%. Even in this case, a meta-regression analysis with follow-up duration as covariate was performed in the attempt to explain the heterogeneity; the no significance was observed (*p* = 0.177).

**Fig. 5:**
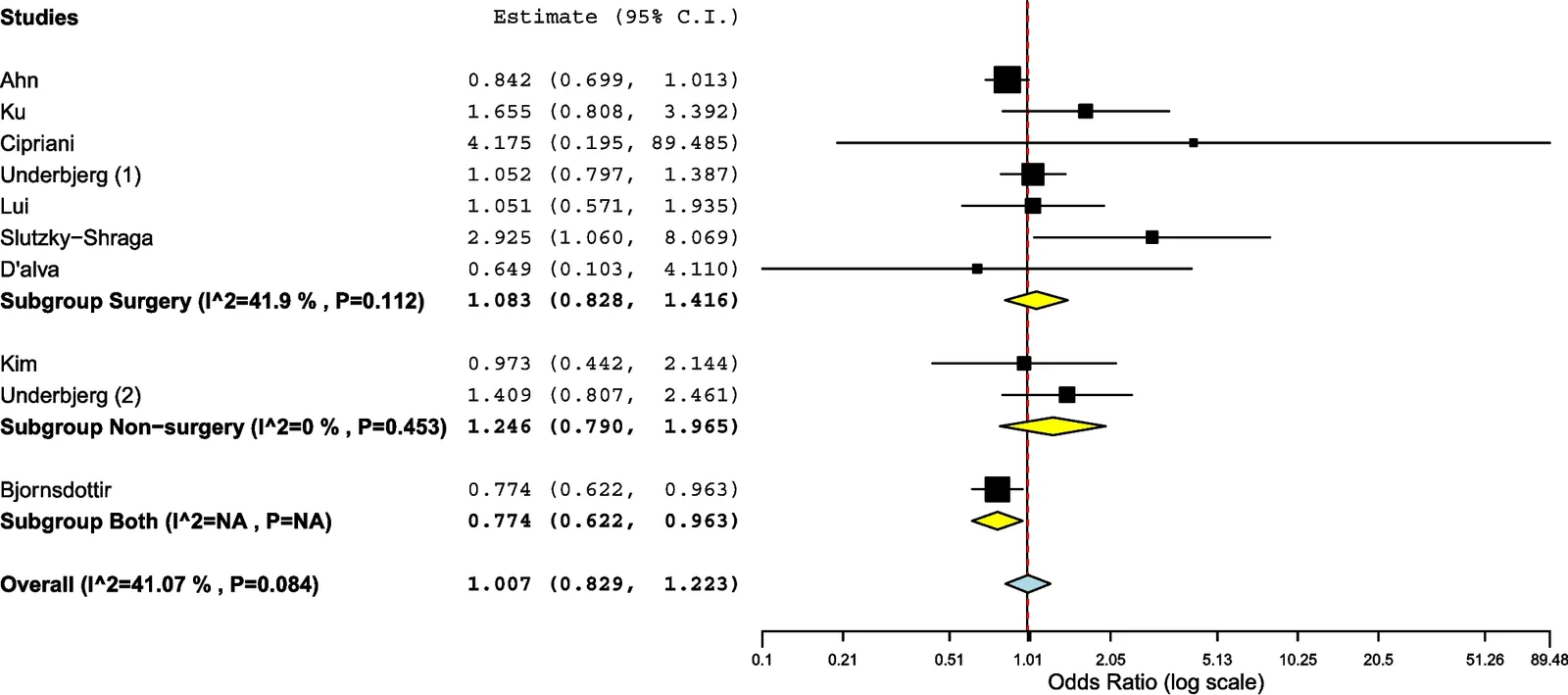
Non-vertebral fractures: OR comparing HypoPT and controls. Blue diamond indicates the pooled OR in all included studies and its wideness indicates 95%CI. Yellow diamonds indicate the pooled OR in subgroups. Black square and black line of any series indicate its weight and 95% CI, respectively

No significant difference in the risk of non-vertebral fractures was observed between patients with HypoPT and controls (OR 1.01 (95% CI 0.83, 1.22), with low heterogeneity across studies (I^2^ = 41.07%) (Fig. 5).

#### Risk of hip Fracture

**Fig. 6:**
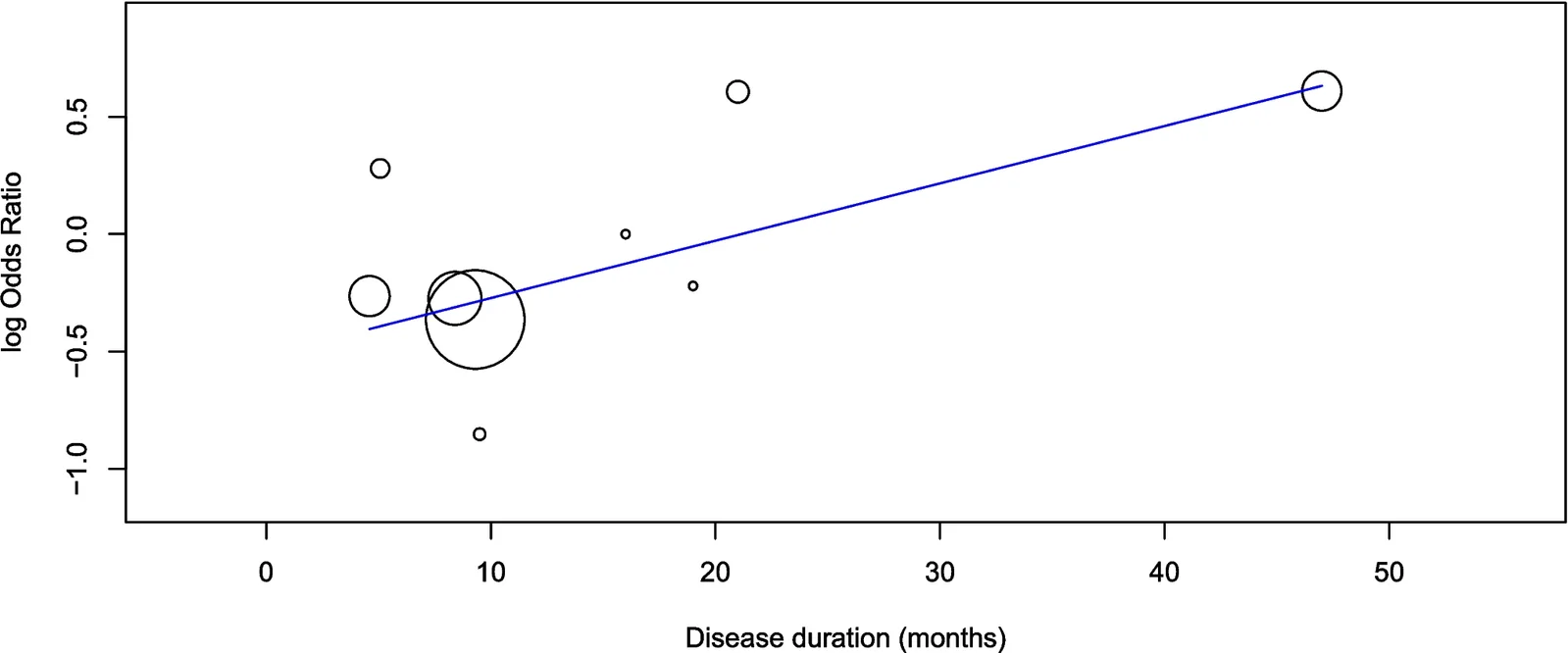
Hip fractures: meta-regression analysis exploring OR comparing HypoPT and controls according to disease duration. Any circle identifies one series, and its size differs according to the series weight

Nine studies [2, 4, 6, 8–10, 13, 15, 18] investigated the percentage of hip fracture in 12,394 HypoPT cases and 128,598 controls. The total number of HypoPT with hip fractures was 73/12,394, while the number of controls with hip fractures was 638/128,598. The pooled proportion of hip fracture was 0.009 (95% CI 0.002–0.016) with I^2^ 74.97% in HypoPT group. This heterogeneity was explained by metaregression analysis showing that the longer is the follow-up the higher the proportion of fracture (*p* = 0.03) (Fig. 6). The proportion of hip fracture in control group was 0.015 (95% CI 0.005–0.025) with I^2^ 98.45%.

**Fig. 7:**
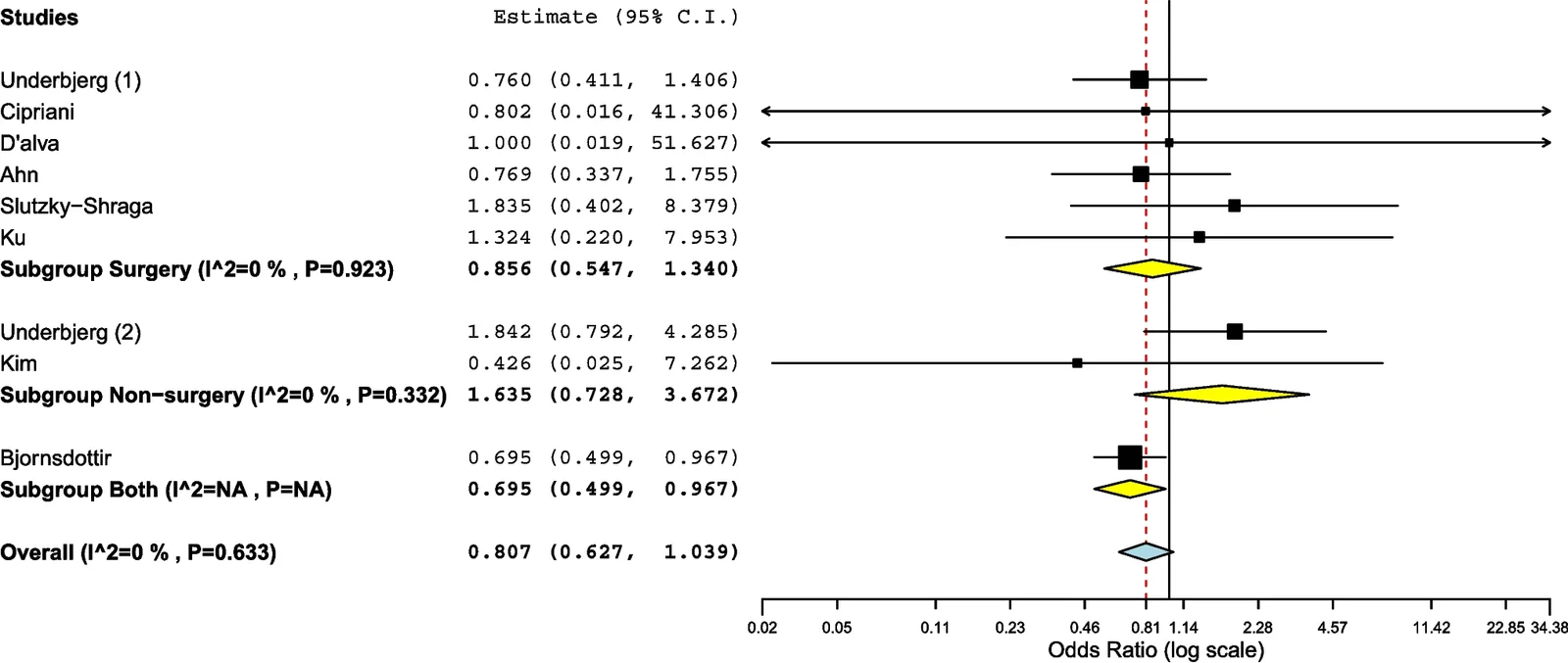
Hip fractures: OR comparing HypoPT and controls. Blue diamond indicates the pooled OR in all included studies and its wideness indicates 95%CI. Yellow diamonds indicate the pooled OR in subgroups. Black square and black line of any series indicate its weight and 95% CI, respectively

No significant difference in the risk of hip fractures was observed between subjects with HypoPT and controls (OR 0.807 [95% CI 0.627–1.039] without heterogeneity [I^2^ 0%]) (Fig. 7).

## Discussion

In this systematic review and meta-analysis, we provide a comprehensive synthesis of the available evidence on fracture risk in patients with chronic HypoPT. The main finding is a site-specific increase in VFs risk, consistently observed in patients with non-surgical HypoPT, whereas no clear increase in risk emerged for post-surgical disease. In contrast, no overall increase in non-vertebral or femoral fracture risk was observed in the overall HypoPT population. Importantly, meta-regression analyses indicated that longer median disease duration was associated with a higher fracture prevalence at non-vertebral and femoral sites, suggesting a progressive increase in risk over time, although the available data do not allow the identification of a precise temporal threshold.

Our findings are consistent with several observational studies reporting an increased prevalence or incidence of VFs despite normal or elevated BMD. This apparent paradox is particularly evident in cohorts of non-surgical HypoPT, including idiopathic and genetically determined forms. Long-term follow-up data in patients with idiopathic HypoPT have demonstrated a higher prevalence of morphometric VFs compared with controls, even in relatively young individuals and despite increased BMD at the lumbar spine and hip [17]. Notably, VFs in these cohorts were frequently clinically silent, underscoring the risk of underdiagnosis when fracture assessment relies exclusively on clinical events.

Population-based data from Korea provide robust evidence of an increased VFs risk in patients with non-surgical HypoPT [15]. In a nationwide matched cohort study, nonsurgical HypoPT was associated with a more than two-fold higher risk of VFs compared with controls, whereas fracture risk at other skeletal sites was variably affected [15]. Similarly, additional observational studies have reported a higher prevalence of VFs in non-surgical HypoPT despite increased BMD [5] particularly among postmenopausal women [17]. Danish nationwide data also suggest an overall increased fracture burden in non-surgical HypoPT, although vertebral fractures may be underestimated due to reliance on hospital discharge diagnoses [6].

Recently, detailed phenotyping studies integrating VFs assessment with trabecular bone score (TBS) have provided additional mechanistic insights. In cohorts of chronic HypoPT, a high prevalence of vertebral fractures has been observed alongside relatively preserved or elevated BMD but impaired trabecular microarchitecture, as reflected by abnormal TBS values [4, 5]. Increased VFs risk occurs despite non-significant increased risk at femoral and non-vertebral fractures. This pattern suggests that low bone turnover in HypoPT differentially affects trabecular-rich vertebral bodies versus cortical-predominant long bones [1]. The suppressed bone remodeling leads to increased trabecular volume and width but may result in accumulation of microdamage and degraded microarchitecture particularly in trabecular bone, rendering vertebrae more susceptible to compression fractures. Over time, these changes may render bone stiffer but more brittle, disproportionately affecting trabecular-rich sites such as the spine [1]. Conversely, the cortical bone in long bones may benefit from reduced remodeling through decreased porosity, explaining the protective effect at appendicular sites.

In contrast, no significant increase in fracture risk was observed in postsurgical HypoPT although the available data remains more heterogeneous. Several large registry-based studies, particularly those including patients undergoing thyroidectomy for benign or malignant disease, reported no overall increase in fracture risk and, in some cases, even a reduced risk of fractures at cortical sites such as the hip, humerus, or wrist [8, 9, 14]. These findings have been interpreted as reflecting the low bone turnover state induced by PTH deficiency, which may confer relative protection against cortical bone loss.

On the other hand, smaller clinical studies and imaging-based investigations challenge the assumption that postsurgical HypoPT is uniformly protective against skeletal fragility. Cross-sectional studies using VFs assessment or morphometric techniques have demonstrated a substantial burden of vertebral fractures in postmenopausal women with chronic postsurgical HypoPT, despite preserved or increased BMD [3, 4, 18]. Similarly, single-center cohorts with long-term follow-up reported that VFs represent the most frequent fracture type in postsurgical HypoPT, often occurring as nonclinical morphometric fractures [13].

The apparent discrepancy between large administrative database studies and smaller imaging-based cohorts likely reflects differences in fracture ascertainment. Registry studies typically capture clinically overt fractures requiring hospitalization, whereas VFs, especially low-grade morphometric fractures, are frequently underrecognized unless systematically assessed. This methodological limitation may lead to an underestimation of VFs burden in postsurgical HypoPT in population-based datasets.

Moreover, our meta-regression analysis indicates that fracture risk of HypoPT patients tends to increase with longer disease duration, supporting a progressive, time-dependent effect of chronic PTH deficiency on skeletal fragility, although no precise temporal threshold could be identified. This mechanistic model predicts increased vertebral risk alongside decreased or neutral appendicular fracture risk, precisely matching the observed pattern across all studies. The potential association between fracture risk and disease duration observed in long-term cohorts of idiopathic HypoPT, as well as in registry studies with extended follow-up, supports the concept that skeletal fragility in HypoPT is a dynamic process that evolves over years rather than an immediate consequence of diagnosis. Although the overall risk at these sites was not significantly increased, this finding may potentially support the concept that prolonged suppression of bone remodeling may progressively impair bone quality over time.

Our findings have important clinical implications. First, they support a site-specific approach to fracture risk assessment in HypoPT, with particular attention to VFs. Second, they suggest that patients with non-surgical HypoPT represent subgroups at higher skeletal risk including an overall risk of any fractures. Routine reliance on BMD alone may be insufficient in these patients, and consideration should be given to vertebral imaging [19]. The potential role of additional clinical risk factors for fractures (e.g., menopausal status, concomitant medications, or biochemical control) could not be assessed due to the lack of consistent reporting across the included studies.

### Strengths and limitations

For this meta-analysis several limitations must be acknowledged. The included studies were observational in nature and heterogeneous with respect to fracture definitions, ascertainment methods, and duration of follow-up. In particular, registry-based studies primarily captured clinically overt fractures identified through hospital discharge codes, whereas morphometric VFs were systematically assessed only in smaller imaging-based cohorts. This discrepancy likely results in an underestimation of VFs burden in large administrative datasets. Another limitation is that two included studies did not clearly distinguish between the number of fractures and the number of patients with fractures, which may affect estimated prevalences [8, 9]. In addition, individual patient-level data were not available, precluding more granular analyses of biochemical control, treatment regimens, recombinant PTH use, and temporal fracture trajectories.

The strengths of this meta-analysis include the comprehensive inclusion of population-based cohorts, single-center studies, and imaging-based investigations, allowing integration of complementary perspectives on fracture risk in chronic HypoPT. Importantly, compared with the previous systematic review and meta-analysis published in 2021 [7] the present work substantially expands the available evidence base. While the earlier meta-analysis included only seven observational studies with a total of approximately 1,500 hypoparathyroid patients, predominantly derived from small cohorts and case, control designs, the current meta-analysis incorporates data from large, population-based cohort studies published after 2021. These studies [8–10, 14, 16] collectively include several thousand patients with chronic HypoPT, the majority of whom had postsurgical disease following thyroidectomy. The inclusion of these large nationwide cohorts markedly increases statistical power, improves the generalizability of the findings, and allows a more robust evaluation of fracture risk in postsurgical HypoPT, a subgroup that was underrepresented and inconclusively characterized in earlier pooled analyses.

In addition, the newly included population-based studies provide site-specific fracture data and extended follow-up durations, enabling a more refined assessment of vertebral versus non-VFs risk patterns. This expanded evidence base allows the present meta-analysis to move beyond the dichotomous conclusion of increased fracture risk limited to nonsurgical HypoPT and to highlight the modifying role of disease duration across etiological subgroups.

Furthemore, compared with the previous meta-analysis, despite including the same studies, we found a significantly increased risk of any fracture in patients with non-surgical HypoPT compared with controls. This difference is likely attributable to methodological discrepancies. Specifically, the previous analysis was based on the total number of fracture events, whereas our approach considered only the number of patients experiencing at least one fracture, thereby reducing the potential bias introduced by individuals with multiple fracture events.

## Conclusions

In conclusion, this meta-analysis demonstrates that chronic HypoPT is associated with a site-specific increase in VFs risk, particularly in patients with non-surgical disease, while no overall increase in non-vertebral or femoral fracture risk was observed. Disease duration appears to modulate fracture prevalence, supporting the concept of cumulative skeletal impairment over time. These findings highlight the need for longitudinal and site-specific fracture assessment and underscore that skeletal fragility in HypoPT cannot be adequately captured by bone density measurements alone. Further prospective studies are warranted to define optimal strategies for fracture prevention and monitoring in this complex endocrine disorder.

## Supplementary Information

Below is the link to the electronic supplementary material.

Supplementary file1 (DOCX 20 KB)

## Data Availability

No datasets were generated or analysed during the current study.

## Abbreviations

BMD: Bone mineral density
HypoPT: Hypoparathyroidism
OR: Odds ratio
PTH: Parathyroid hormone
APECED: Polyendocrinopathy–candidiasis–ectodermal dystrophy
RANKL: Receptor Activator of Nuclear factor Kappa-B Ligand
TBS: Trabecular bone score
VFs: Vertebral fractures
