# Supplementary material for: Site-specific fracture risk in chronic hypoparathyroidism: systematic review and meta-analysis
Source: Rev Endocr Metab Disord. 2026 May 11;27(4):837–52. doi: 10.1007/s11154-026-10051-8 (PMC13653866; doi:10.1007/s11154-026-10051-8)
Supplement: Supplementary file 1 [file 11154_2026_10051_MOESM1_ESM.docx]

**Table S1.** Risk of bias summary: review of authors’ judgements about each risk of bias item for each included in the meta-analysis.

| **Author** | **Year** | **Q1** | **Q2** | **Q3** | **Q4** | **Q5** | **Q6** | **Q7** | **Q8** | **Q9** | **Q10** | **Q11** | **Q12** | **Q13** | **Q14** | **High_risk_n** | **NR_n** | **Overall_quality** |
| --- | --- | --- | --- | --- | --- | --- | --- | --- | --- | --- | --- | --- | --- | --- | --- | --- | --- | --- |
| Reddy Nl | 2025 | L | L | NR | L | H | L | H | NR | L | L | L | H | NR | H | 4 | 3 | POOR |
| Mendonça ml | 2013 | L | L | NR | L | L | L | L | NR | L | L | L | H | NR | L | 1 | 3 | FAIR |
| Saha | 2022 | L | L | NR | L | H | L | L | NR | L | L | L | H | NR | L | 2 | 3 | FAIR |
| Slutzky-Shraga I | 2024 | L | L | NR | L | H | L | L | NR | L | L | L | H | NR | L | 2 | 3 | FAIR |
| Vadiveloo T | 2018 | L | L | NR | L | H | L | L | L | L | L | L | H | NR | H | 3 | 3 | POOR |
| Brasil d’Alva C | 2022 | L | L | NR | L | H | L | L | NR | L | L | L | H | L | H | 3 | 2 | POOR |
| David Tak Wai Lui | 2021 | L | L | L | L | H | L | L | NR | L | L | L | H | NR | L | 2 | 2 | FAIR |
| Cipriani C | 2021 | L | L | NR | L | H | L | L | NR | L | L | L | H | NR | L | 2 | 3 | FAIR |
| Underbjerg L | 2014 | L | L | NR | L | H | L | L | NR | L | L | L | H | NR | L | 2 | 3 | FAIR |
| Underbjerg L | 2015 | L | L | NR | L | H | L | L | NR | L | L | L | H | NR | H | 3 | 3 | POOR |
| Björnsdottir B | 2025 | L | L | NR | L | L | L | L | L | L | L | L | H | NR | L | 1 | 2 | FAIR |
| Seong Hee Ahn | 2023 | L | L | NR | L | H | L | L | NR | L | L | L | H | NR | L | 2 | 3 | FAIR |
| Chawla H | 2017 | L | L | NR | L | H | L | L | NR | L | L | L | H | NR | L | 2 | 3 | FAIR |
| Ku EJ | 2025 | L | L | NR | L | H | L | H | NR | L | L | L | H | NR | L | 3 | 3 | POOR |
| Se Hwa Kim | 2020 | L | L | NR | L | H | L | L | NR | L | L | L | H | NR | L | 2 | 3 | FAIR |

L, low risk; H, high risk; N.R., not reported.

**Questions:**

1. Research question clearly stated?

2. Study population clearly specified?

3. Participation rate ≥50%?

4. Groups recruited from same population?

5. Sample size justification provided?

6. Exposure measured prior to outcome?

7. Sufficient timeframe to see effect?

8. Different levels of exposure examined?

9. Exposure measures clearly defined?

10. Exposure assessed consistently?

11. Outcome measures clearly defined?

12. Outcome assessors blinded?

13. Loss to follow-up ≤20%?

14. Confounders measured and adjusted?
